# Effect of intracoronary agents on the no-reflow phenomenon during primary percutaneous coronary intervention in patients with ST-elevation myocardial infarction: a network meta-analysis

**DOI:** 10.1186/s12872-017-0722-z

**Published:** 2018-01-10

**Authors:** Xiaowei Niu, Jingjing Zhang, Ming Bai, Yu Peng, Shaobo Sun, Zheng Zhang

**Affiliations:** 1 The First School of Clinical Medicine, Lanzhou University, Tianshui South Road, No. 222, Lanzhou, Gansu 730000 China; 2 Baiyin Second People’s Hospital, Gongyuan Road, No. 509, Baiyin, Gansu 730900 China; 3 Department of Cardiology, the First Hospital of Lanzhou University, Donggang West Road, No. 1, Lanzhou, Gansu 730000 China; 4 Key Lab of Prevention and Treatment for Chronic Disease, Gansu University of Chinese Medicine, Dingxi East Road, No. 35, Lanzhou, Gansu 730000 China

**Keywords:** Percutaneous coronary intervention, No reflow phenomenon, Intracoronary; pharmacology, Meta-analysis

## Abstract

**Background:**

Despite the restoration of epicardial flow after primary percutaneous coronary intervention (PPCI), myocardial reperfusion remains impaired in a significant proportion of patients. We performed a network meta-analysis to assess the effect of 7 intracoronary agents (adenosine, anisodamine, diltiazem, nicorandil, nitroprusside, urapidil, and verapamil) on the no-reflow phenomenon in patients with ST-elevation myocardial infarction (STEMI) undergoing PPCI.

**Methods:**

Database searches were conducted to identify randomized controlled trials (RCTs) comparing the 7 agents with each other or with standard PPCI. Outcome measures included thrombolysis in myocardial infarction flow grade (TFG), ST-segment resolution (STR), left ventricular ejection fraction (LVEF), major adverse cardiovascular events (MACEs), and adverse events.

**Results:**

Forty-one RCTs involving 4069 patients were analyzed. The addition of anisodamine to standard PPCI for STEMI was associated with improved post-procedural TFG, more occurrences of STR, and improvement of LVEF. The cardioprotective effect of anisodamine conferred a MACE-free survival benefit. Additionally, nitroprusside was regarded as efficient in improving coronary flow and clinical outcomes. Compared with standard care, adenosine, nicorandil, and verapamil improved coronary flow but had no corresponding benefits regarding cardiac function and clinical outcomes. The ranking probability for the 7 treatment drugs showed that anisodamine consistently ranked the highest in efficacy outcomes (TFG < 3, STR, LVEF, and MACEs). No severe adverse events, such as hypotension and malignant arrhythmia, were observed in patients treated with anisodamine. Network meta-regression analysis showed that age, the time to reperfusion, and study follow-up did not affect the treatment effects.

**Conclusions:**

The intracoronary administration of anisodamine appears to improve myocardial reperfusion, cardiac function, and clinical outcomes in patients with STEMI undergoing PPCI. Given the limited quality and quantity of the included studies, more rigorous RCTs are needed to verify the role of this inexpensive and well-tolerated regimen.

**Electronic supplementary material:**

The online version of this article (10.1186/s12872-017-0722-z) contains supplementary material, which is available to authorized users.

## Background

Primary percutaneous coronary intervention (PPCI) is the preferred reperfusion therapy for ST-elevation myocardial infarction (STEMI) [1]. Despite the restoration of epicardial flow after PPCI, impaired myocardial perfusion, known as the no-reflow phenomenon (NR), remains observed in a significant proportion of patients [2]. The NR after PPCI for the treatment of STEMI contributes to infarct size expansion, reduced ventricular function, and increased mortality [3–5]. Several potential mechanisms have been hypothesized to cause NR, including embolization of atherothrombotic material, vasoconstriction, activation of the inflammatory cascade, neutrophil plugging, platelet aggregation, toxic free-radical generation, and myocardial edema [2, 6]. A basic understanding of the process has contributed to several pharmacological drugs proposed to improve myocardial reperfusion after PPCI, such as adenosine, diltiazem, nicorandil, nitroprusside, urapidil, and verapamil [2, 6]. A number of meta-analyses have been conducted to assess whether intracoronary adenosine, diltiazem, nitroprusside, nicorandil, and verapamil can reduce NR and improve clinical outcomes after PPCI. However, these meta-analyses were limited in size and yielded inconclusive results [7]. After the most recent meta-analyses were published [8–12], new randomized controlled trials (RCTs) on this subject have been published, and these additional data may help reduce the amount of uncertainty surrounding the treatment effects. Anisodamine, unlike the aforementioned vasodilators, is a muscarinic cholinergic antagonist [13]. Basic and clinical studies have shown that anisodamine can increase blood pressure and coronary perfusion pressure and improve microcirculation, making it a potentially useful drug for preventing NR [14–16]. However, the value of anisodamine in improving myocardial reperfusion after PPCI has not been studied in any previous meta-analysis.

Traditional pairwise meta-analyses are limited for simultaneously synthesizing all evidence because head-to-head comparisons between treatments are often unavailable [17]. Bayesian network meta-analysis combines direct and indirect comparisons and forms hierarchies for the efficacy of various treatments [18]. Thus, this technique better informs clinicians regarding the optimal use of candidate agents in clinical practice. In this study, we performed standard pairwise and Bayesian network meta-analyses to comprehensively evaluate available intracoronary agents as adjuncts to PPCI, to estimate the relative efficacy and safety of the various agents, and to provide a hierarchy of treatments for the outcomes of interest.

## Methods

### Study search strategy

PubMed, Embase, Web of Science, and CENTRAL databases were searched systematically, as well as the references of eligible studies and recent reviews from inception to December 31, 2016. The keywords and corresponding Medical Subject Headings (Mesh) were as follows: “adenosine”, “anisodamine”, “calcium channel blockers”, “diltiazem”, “verapamil”, “nicorandil”, “nitroprusside”, “urapidil”, “vasodilator agents”, “intracoronary”, “myocardial infarction”, and “percutaneous coronary intervention”. No limits regarding language and publication type were applied.

### Selection criteria

The inclusion criteria were as follows: (i) RCTs involving patients with STEMI undergoing PPCI; (ii) studies that evaluated intracoronary administration of any of the drugs (adenosine, anisodamine, diltiazem, verapamil, nicorandil, nitroprusside, and urapidil) and compared the active drugs to each other or a control (standard PPCI without the use of the aforementioned active drugs); and (iii) trials that reported data on any of the outcomes of interest: thrombolysis in myocardial infarction flow grade (TFG) < 3 [19], ST-segment resolution (STR) defined as at least a 50–70% resolution of ST-segment elevation on an electrocardiogram after PPCI compared with the baseline measurement [20], left ventricular ejection fraction (LVEF), major adverse cardiovascular events (MACEs), and adverse events (AEs). Indicators of reperfusion (TFG and STR) were measured after PPCI. For LVEF outcome, we pooled the data assessed in-hospital to 1 month after PPCI because the data were available in most studies. MACEs was evaluated at the longest available follow-up. The exclusion criteria were as follows: (i) trials containing only one of the aforementioned treatments, (ii) duplicate reporting, and (iii) sub-studies of the RCTs.

### Data collection and quality assessment

On the basis of the title, abstract, or full-texts, two independent investigators assessed the studies for eligibility in three screening stages and then extracted data based on the pre-specified forms. The following information was included: (i) the designs of trials and inclusion criteria, (ii) patient characteristics at baseline, (iii) features of the interventions, and (iv) outcomes as aforementioned. Different reviewers independently assessed the methodological quality of eligible trials using the criteria of the Cochrane Handbook [21]. For missing or unclear information, we attempted to contact the original trial authors by e-mail. All divergences were resolved by consensus or adjudication by a third reviewer.

### Statistical analysis

Two investigators cross-checked the data from all the identified studies. Standard pairwise and network meta-analyses were performed to obtain estimates for outcomes, and these estimates were presented as odds ratios (ORs) or mean differences (MDs) with 95% CIs for dichotomous or continuous data, respectively. First, we conducted the standard pairwise meta-analysis using STATA 11.0 software (Stata Corp., College Station, Texas, USA). A random-effect model was preferred because of the anticipated variety in study populations. Statistical heterogeneity was evaluated using the Cochrane Q test and *I*^2^ statistic (*I*^2^ values >75% represented significant heterogeneity). Funnel plots were used to evaluate the publication bias of each endpoint. A 0.5 zero-cell correction was used so that studies with no events would still be included for analyses [21]. Second, we performed Bayesian network meta-analysis and meta-regression using WinBUGS 1.4.3 software (MRC Biostatistics Unit, Cambridge, UK) [22]. A random-effect model was used to compare treatments using Markov chain Monte Carlo methods with Gibbs sampling from 150,000 iterations obtained after a 90,000 iteration burn-in phase. Model convergence was assessed graphically according to Gelman and Rubin. Model fit was evaluated by comparing a posterior mean residual deviance to the number of independent data points. The inconsistency of the network was assessed by contrasting estimates from the network analysis with the direct comparison meta-analysis of each node (node splitting). A Bayesian *P* value >0.05 from the method represented the presence of consistency between direct and indirect comparisons. In addition, we used the surface under the cumulative ranking curve (SUCRA) to rank the treatments for each outcome. SUCRA expressed as percentages would be 100% when a treatment has a high likelihood of being best and 0% when a treatment has a high likelihood of being worst [18]. Network meta-regression analysis was performed to explore the effects of potential treatment modifying covariates, including mean age, the time to reperfusion, and duration of follow-up. In this analysis, an identical interaction effect across all treatments with respect to the control was assumed [23]. The 95% CIs for the interaction coefficient excluded zero, suggesting an interaction effect between the covariates and the treatment effects [23]. The deviance information criterion (DIC) was used to compare fit between the covariate adjusted and unadjusted models for the same data. Differences in the DIC ≥ 3 were considered meaningful [23]. Results were stratified by the type of control group (placebo or conventional PPCI alone) and median duration of follow-up. Given that MACE definition was not identical in all trials, we performed a sensitivity analysis to evaluate treatments by restricting to studies with a relatively uniform definition of MACEs. Data plotting was performed using R 3.3.1 software (R Core Team, Vienna, Austria) [24]. The results were statistically significant with a two-sided *P* < 0.05. We performed this meta-analysis according to the Preferred Reporting Items for Systematic Reviews and Meta-Analyses (PRISMA) statement and Cochrane Handbook guidelines [21, 25].

## Results

### Study selection and characteristics of the included trials

**Fig. 1:**
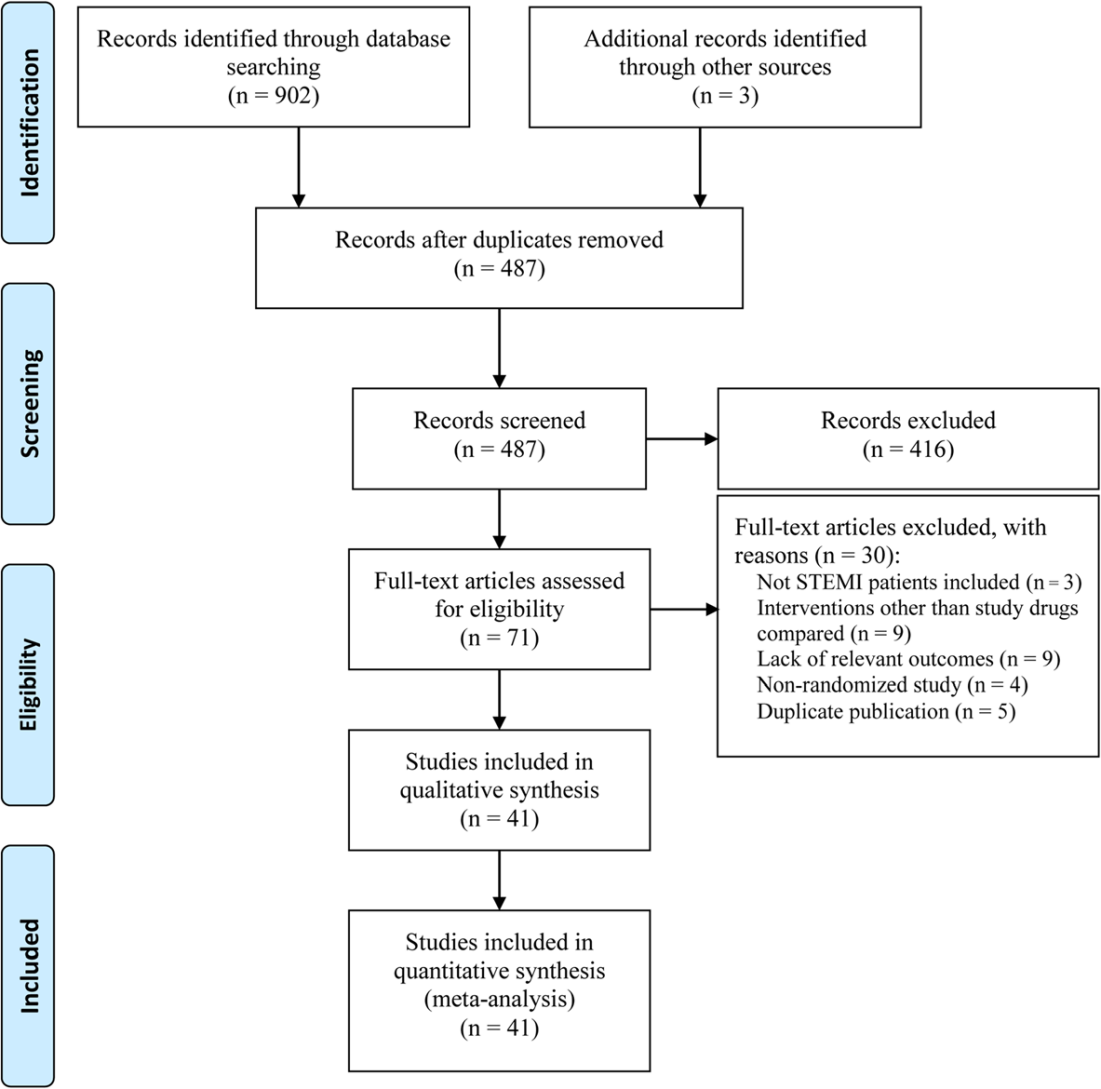
Flow chart of study selection

**Fig. 2:**
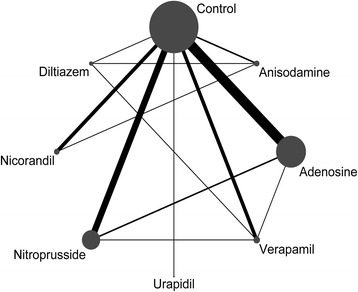
Network of treatment comparisons. Nodes and edges are weighted according to the number of patients who received each treatment and direct comparison, respectively

Of 905 potentially eligible trials, 41 RCTs involving 4069 patients were included in this network meta-analysis (Fig. 1) [26–66]. Seven drug classes were administered intracoronarily during PPCI, including adenosine (*n* = 1006), anisodamine (*n* = 208), diltiazem (*n* = 88), verapamil (*n* = 181), nicorandil (*n* = 217), nitroprusside (*n* = 551), and urapidil (*n* = 49). The network of available treatment comparisons is shown in Fig. 2. Nineteen studies used placebo (saline solution) as a comparator, whereas 20 studies used conventional PPCI alone as the comparator. Participants were enrolled at a mean age of 61 years, and male participants accounted for 78% of the total population. The time to reperfusion from the onset of symptoms ranged from 116 min to 546 min across studies and the median time to reperfusion across all studies was 273 min. The proportion of patients with multivessel disease ranged from 15 to 65% across 14 studies and the median proportion of patients with multivessel disease across 14 studies was 52% [30, 36–38, 43–45, 50, 51, 54, 55, 59, 61, 65]. Twenty-seven studies reported the incidence of MACEs within a 6-month follow-up, one trial reported within an 8-month follow-up, and four trials reported within a 12-month follow-up. Studies that evaluated adenosine, anisodamine, diltiazem, verapamil, nicorandil, nitroprusside, and urapidil treatments had median follow-up periods of 6 months, 4.5 months, 3.5 months, 1 month, 2 months, 6 months, and 1 month, respectively. Eight RCTs used the relatively uniform definition of MACEs including death, reinfarction, and revascularization. The main characteristics of the identified trials are detailed in Table 1. A summary of outcomes for each agent is listed in Table 2.

**Table 1:**
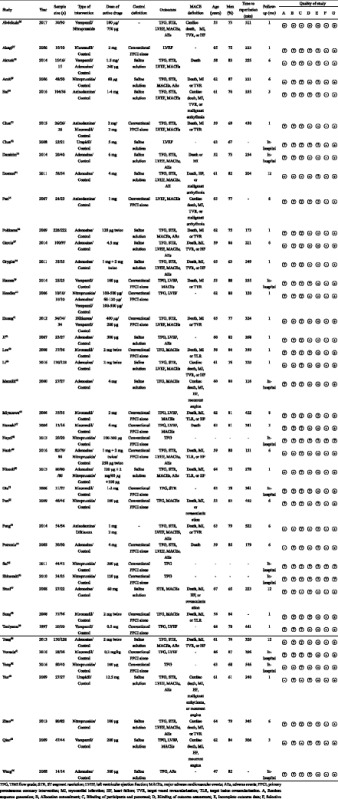
Description of included studies

**Table 2:** Summary of outcomes for each agent

Type of intervention	Outcomes										
	TFG < 3		STR		LVEF			MACEs		AEs	
	Events	Total	Events	Total	Mean	SD	Total	Events	Total	Events	Total
Adenosine	82	978	538	931	48.80	11.72	502	115	959	258	796
Anisodamine	18	184	140	184	58.78	10.31	208	11	208	29	158
Diltiazem	20	88	43	88	54.21	12.33	88	12	88	19	54
Nicorandil	7	207	33	57	55.18	11.75	112	20	148	–	–
Nitroprusside	57	551	191	317	51.46	12.44	199	26	363	50	317
Urapidil	1	27	25	27	54.32	6.65	49	0	27	4	27
Verapamil	26	181	34	79	52.56	12.48	181	18	151	3	30
Control	263	1693	608	1219	50.57	10.88	943	228	1478	87	1008

TFG, TIMI flow grade; STR, ST-segment resolution; LVEF, left ventricular ejection fraction; MACEs, major adverse cardiovascular events; AEs, adverse events

Overall, the methodological quality of the included trials was not high (Table 1). The risk of bias was low for random sequence generation in 13 trials (32%), allocation concealment in 8 trials (20%), blinding of participants and personnel in 5 trials (12%), blinding of outcome assessment in 24 trials (59%), incomplete outcome data in 25 trials (61%), selective reporting in 38 trials (93%), and other sources of bias in 38 trials (93%).

### TFG < 3

**Fig. 3:**
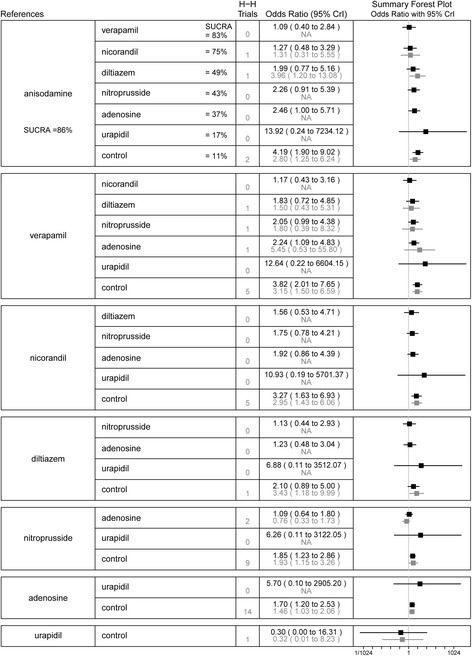
Summary forest plot table for the outcome of thrombolysis in myocardial infarction flow grade < 3. H-H trials, head-to-head trials; black text, network meta-analysis results; grey text, pairwise meta-analysis results; CrI, credible interval; SUCRA, the surface under the cumulative ranking curve; Interventions are displayed by SUCRA percentages; Each intervention in the second column was compared with the intervention listed in the first column

In the network meta-analysis, 37 studies with 3909 patients contributed to the analysis of TFG < 3 after PPCI. The incidence of TFG < 3 was higher in the control group than in the anisodamine (OR 4.19, 95%CI 1.90–9.02), verapamil (OR 3.82, 95%CI 2.01–7.65), nicorandil (OR 3.27, 95%CI 1.63–6.93), nitroprusside (OR 1.85, 95%CI 1.23–2.86), and adenosine (OR 1.46, 95%CI 1.03–2.06) groups. Adenosine was associated with a significantly increased risk of TFG < 3 compared with anisodamine (OR 2.46, 95%CI 1.00–5.71) and verapamil (OR 2.24, 95%CI 1.09–4.83). In the SUCRA analysis, the hierarchy for treatment efficacy for TFG < 3 (highest to lowest rank) was anisodamine, followed by verapamil, nicorandil, diltiazem, nitroprusside, adenosine, urapidil, and the control. In the traditional pairwise meta-analysis, there was no significant heterogeneity for any treatment effect across strata (all *I*^2^ < 75%; *P*_het_ > 0.10). Similar results were observed between the control group and the adenosine (OR 1.46, 95%CI 1.03–2.06), anisodamine (OR 2.80, 95%CI 1.25–6.24), nicorandil (OR 2.95, 95%CI 1.43–6.06), nitroprusside (OR 1.93, 95%CI 1.15–3.26), and verapamil (OR 3.15, 95%CI 1.50–6.59) groups. All comparisons analyzed in the network and pairwise meta-analyses are shown in Fig. 3.

### STR

**Fig. 4:**
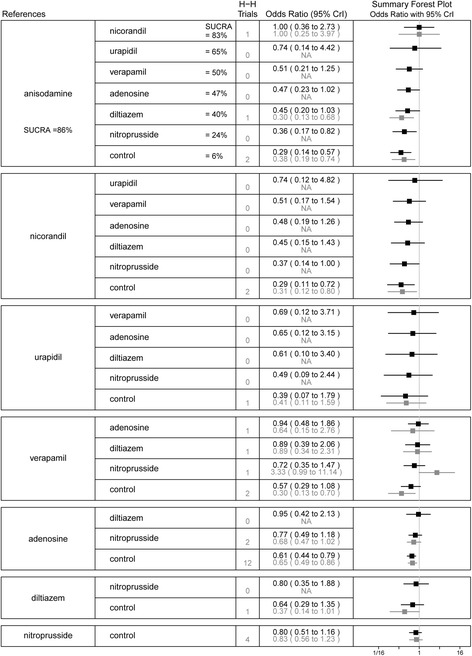
Summary forest plot table for the outcome of ST-segment resolution. H-H trials, head-to-head trials; black text, network meta-analysis results; grey text, pairwise meta-analysis results; CrI, credible interval; SUCRA, the surface under the cumulative ranking curve; Interventions are displayed by SUCRA percentages; Each intervention in the second column was compared with the intervention listed in the first column

Twenty-one studies with 2902 patients contributed to the evidence network for STR analysis. The control group was associated with a lower rate of STR compared with the anisodamine (OR 0.29, 95%CI 0.14–0.57), nicorandil (OR 0.29, 95%CI 0.11–0.72), and adenosine (OR 0.61, 95%CI 0.44–0.79) groups. Nitroprusside was associated with a significantly reduced rate of STR compared with anisodamine (OR 0.36, 95%CI 0.17–0.82) and nicorandil (OR 0.37, 95%CI 0.14–1.00). As indicated by the SUCRA value, anisodamine ranked the highest, and the control ranked the lowest, indicating that anisodamine was most likely to be the best treatments for this outcome. For pairwise meta-analysis, results of the comparison between the control group and the adenosine (OR 0.65, 95%CI 0.49–0.86), anisodamine (OR 0.38, 95%CI 0.19–0.74), and nicorandil (OR 0.31, 95%CI 0.12–0.80) groups were similar to those of the network meta-analysis. There was no significant heterogeneity for any treatment effect across strata (all *I*^2^ < 75%; *P*_het_ > 0.05). The network and pairwise treatment comparisons are shown in Fig. 4.

### LVEF

**Fig. 5:**
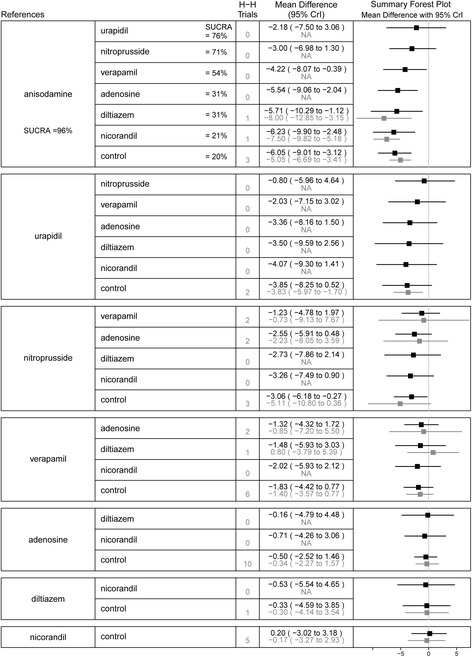
Summary forest plot table for the outcome of left ventricular ejection fraction. H-H trials, head-to-head trials; black text, network meta-analysis results; grey text, pairwise meta-analysis results; CrI, credible interval; SUCRA, the surface under the cumulative ranking curve; Interventions are displayed by SUCRA percentages; Each intervention in the second column was compared with the intervention listed in the first column

Twenty-seven studies with 2282 patients were involved in the network meta-analysis for LVEF. Standard care had a significantly lower LVEF after PPCI compared to anisodamine (MD -6.05, 95%CI -9.01 to -3.12) and nitroprusside (MD -3.06, 95%CI -6.18 to -0.27) therapy. Compared to the anisodamine group, the verapamil (MD -4.22, 95%CI -8.07 to -0.39), adenosine (MD -5.54, 95%CI -9.06 to -2.04), diltiazem (MD -5.71, 95%CI -10.29 to-1.12), and nicorandil (MD -6.23, 95%CI -9.90 to -2.48) groups had a significantly lower LVEF. Results of the SUCRA analysis showed that anisodamine was most possibly the best treatment, whereas the control was rated as the least effective treatment. Similarly, results of the pairwise meta-analysis showed that standard care had a lower LVEF than anisodamine (MD -5.05, 95%CI -6.69 to -3.41). However, there was a statistically significant difference in LVEF between the control and urapidil groups (MD -3.83, 95%CI -5.97 to -1.70). Results of the comparison between the control and nitroprusside were no longer significant, but the trend favored nitroprusside. Low heterogeneity was observed for the aforementioned analyses, except for analysis of the control versus nitroprusside (*I*^2^ = 95%; *P*_het_ < 0.05). The network and pairwise treatment comparisons are shown in Fig. 5.

### MACEs

**Fig. 6:**
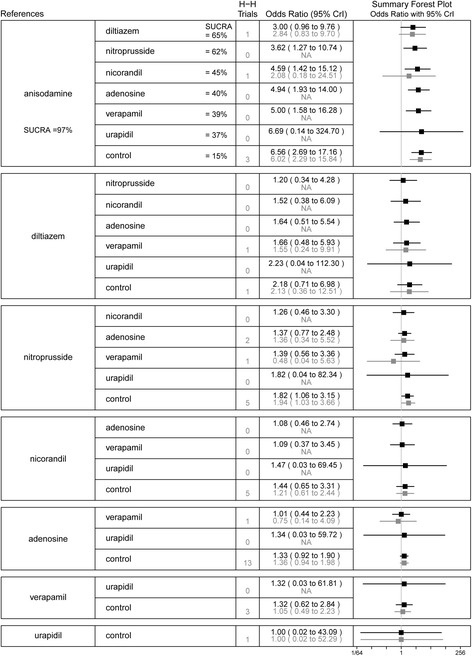
Summary forest plot table for the outcome of major adverse cardiovascular events. H-H trials, head-to-head trials; black text, network meta-analysis results; grey text, pairwise meta-analysis results; CrI, credible interval; SUCRA, the surface under the cumulative ranking curve; Interventions are displayed by SUCRA percentages; Each intervention in the second column was compared with the intervention listed in the first column

Twenty-nine studies with 3422 patients were included in the network meta-analysis for MACEs. Compared with anisodamine, nitroprusside (OR 3.62, 95%CI 1.27–10.74), nicorandil (OR 4.59, 95%CI 1.42–15.12), adenosine (OR 4.94, 95%CI 1.93–14.00), verapamil (OR 5.00, 95%CI 1.58–16.28), and the control (OR 6.56, 95%CI 2.69–17.16) were associated with a significant increase in the risk of MACEs. The incidence of MACEs was higher in the control group than in the nitroprusside group (OR 1.82, 95%CI 1.06–3.15). Results of the SUCRA analysis showed that anisodamine was superior to all other candidate interventions for decreasing the risk of MACEs. In the standard pairwise meta-analysis, we observed similar results between the control and anisodamine (OR 6.02, 95%CI 2.29–15.84), nitroprusside (OR 1.94, 95%CI 1.03–3.66) groups. There was no significant heterogeneity for any treatment effect across strata (all *I*^2^ < 75%; *P*_het_ > 0.05). The network and pairwise treatment comparisons are shown in Fig. 6.

### AEs

AEs were reported in 17 RCTs, including flushing, thirst, hypotension, bradycardia, tachycardia, bronchospasm, atrioventricular block, dyspnea, and chest pain. Fortunately, these AEs were almost always transient, and none of the studies reported any long-lasting sequelae. As there were clear differences regarding the definition of AEs for each individual agent, the network meta-analysis was not conducted. Results of the pairwise meta-analysis showed that adenosine therapy was associated with a higher rate of AEs than control therapy (OR 4.69, 95%CI 1.82–12.08). There was no statistically significant difference in AEs with anisodamine (OR 0.30, 95%CI 0.00–21.04) and nitroprusside (OR 1.23, 95%CI 0.69–2.19) compared with control therapy. There was also no significant between-study heterogeneity for these analyses (all *I*^2^ < 75%; *P*_het_ > 0.05).

### Meta-regression analysis

In network meta-regression analyses (Table 3), we explored the effects of mean age and the time to reperfusion on outcomes: TFG < 3, STR, LVEF, and MACEs, respectively. Since studies reported MACEs at a wide range of follow-up, we also used network meta-regression to adjust for the difference in follow-up periods. The 95% CIs of the interaction coefficients included zero in each adjusted model. None of the covariate adjusted models showed significant improvement in the DIC compared with the unadjusted models.

**Table 3:** Model fit statistics for the covariate adjustment analyses

Covariate adjusted RE Binomial logit model	Statistic	
	Interaction coefficient, median (95% CrI)	DIC difference between adjusted and unadjusted models of the same data
Outcome: TFG < 3		
Age	0.37 (−0.30, 1.05)	0.4
Time of reperfusion	−0.14 (−0.86, 0.71)	1.1
Outcome: STR		
Age	0.24 (−0.49, 0.95)	1.0
Time of reperfusion	0.61 (−0.12, 1.27)	0.1
Outcome: LVEF		
Age	1.88 (−1.59, 5.10)	0.6
Time of reperfusion	3.34 (−1.89, 8.11)	1.0
Follow-up	−0.75 (−3.78, 2.24)	0.5
Outcome: MACEs		
Age	0.15 (−0.71, 1.04)	1.1
Time of reperfusion	−0.49 (−1.41, 0.38)	0.1
Follow-up	0.25 (−0.35, 0.91)	0.5

TFG, TIMI flow grade; STR, ST-segment resolution; LVEF, left ventricular ejection fraction; MACEs, major adverse cardiovascular events; CrI, credible interval; DIC, deviance information criterion; RE, random effect

### Subgroup analysis

For TFG < 3, STR, LVEF, and MACE outcomes, subgroup analysis did not reveal any significant effect modification by the type of control group (all *P* values for interaction > 0.05). For MACE outcome, the analysis stratified by the median duration of follow-up did not yield significantly different results in the two periods (≤ 4.5 months and > 4.5 months). The results of subgroup analysis are presented in Additional file 1: Table S1, Figures S1 and S2.

### Sensitivity analysis

Sensitivity analysis was restricted to studies with the relatively uniform definition of MACEs. The pooled risk estimates in the sensitivity analysis did not change substantially in comparison with the estimates from the main analysis that included all trials reporting MACE outcome (Additional file 1: Figure S3).

### Additional analyses

Visual inspection of the funnel plots did not show asymmetry for any of the analyzed outcomes. The Gelman-Rubin plot confirmed an adequate convergence of the model for each network analysis. Model evaluation indicated adequate fit, as the posterior mean residual deviance was similar to the number of data points in each analysis. There was no evidence of statistical inconsistency between direct and indirect estimates based on the node-splitting method (*P* > 0.05).

## Discussion

Through the present meta-analysis comprising 41 RCTs involving 4069 patients, we found that the addition of anisodamine to standard PPCI for STEMI was associated with improved post-procedural coronary flow, more occurrences of STR, and improvement of LVEF. The cardioprotective effect of anisodamine conferred a MACE-free survival benefit. Among the 7 treatment strategies, anisodamine was superior to the other treatments in efficacy outcomes (TFG < 3, STR, LVEF, and MACEs). Severe or life-threatening AEs were not observed in patients treated with anisodamine. Nitroprusside was also regarded as effective for improving coronary flow and clinical outcomes. Additionally, the intracoronary administration of adenosine, nicorandil, and verapamil exerted some cardioprotection in patients with STEMI undergoing PPCI.

The present network meta-analysis included all available RCTs involving intracoronary agents (adenosine, anisodamine, diltiazem, nicorandil, nitroprusside, urapidil, and verapamil) as adjuncts to PPCI. This study took into account the most recent studies and had the largest sample size to date among meta-analyses assessing the effect of intracoronary agents on NR in patients with STEMI undergoing PPCI. Demonstration of the improvement in indicators of myocardial reperfusion is a key step to the investigation of improved clinical outcomes for intracoronary agents as adjuncts to PPCI [7]. Our network meta-analysis, by evaluating surrogate outcomes (TFG, STR, and LVEF) and the composite clinical endpoint (MACEs), provided a comprehensive insight into the use of 7 intracoronary agents during PPCI. However, most included RCTs were designed to assess angiographic or electrocardiographic outcomes, thus partly leading to a limited number of participants. Although angiographic and electrocardiographic indicators of myocardial reperfusion are well-known prognostic factors [4, 5], the improvement in surrogate markers does not always correspond to improved clinical outcomes. For example, aspiration thrombectomy was considered a simple way to remove the thrombus before stent deployment, thereby improving coronary reperfusion. However, recent trials have demonstrated a lack of benefit of thrombus aspiration on clinical outcomes and suggested possible harm from an increased risk of stroke [67, 68]. Therefore, our finding should be viewed as hypothesis generating, given the limitations of the current available evidence.

Anisodamine, a muscarinic cholinergic antagonist, has been reported with multiple pharmacological effects in basic and clinical studies [13–16, 69, 70]. First, anisodamine inhibits the acetylcholine receptor and modulates the balance between sympathetic and vagus nerve activity during myocardial ischemia/reperfusion [13]. Numerous studies have shown that anisodamine can increase blood pressure and heart rate, and further increase the coronary perfusion pressure [16, 69]. The action of anisodamine is especially appealing, because it can help improve coronary microcirculation and has practical importance. Clinicians often have concerns about hypotension and bradycardia, although they are short-lived after the intracoronary administration of vasodilators. Anisodamine may be a promising drug to address these safety concerns. Second, anisodamine has a similar role to a calcium channel blocker. Anisodamine can prevent intracellular calcium overload, reduce lipid superoxidation, inhibit oxygen free radical formation, and relieve microvascular spasms [70]. Finally, anisodamine decreases post-ischemia/reperfusion myocardial swelling, which can reduce capillary compression from surrounding edematous myocytes [14, 15]. This is the first meta-analysis of RCTs of anisodamine to demonstrate a significant benefit of adjuvant anisodamine over standard care in patients with STEMI undergoing PPCI. Our results showed that anisodamine could significantly improve myocardial reperfusion (reflected by TFG and STR) and cardiac function (reflected by LVEF). Importantly, these effects were translated into improvement of composite clinical outcome (MACEs). Moreover, the analyses of rank probabilities revealed that of 7 treatment strategies, anisodamine consistently ranked the highest in improving TFG, promoting post-procedure STR, ameliorating LVEF, and decreasing the risk of MACEs, which made it the most efficacious drugs according to our results. Therefore, anisodamine may be regarded as an effective, well-tolerated, and possibly cost-effective regimen (currently about a dollar per 10 mg) for prevention of NR. However, it should be noted that this evidence was based on relatively small head-to-head RCTs. Large, high-quality RCTs are needed to fully evaluate the role of anisodamine as an adjunct to reperfusion in patients with STEMI.

Nitroprusside is a direct donor of nitric oxide, which is a potent vasodilator of the resistance arteriolar circulation and has anti-platelet and anti-inflammatory effects [71]. Zhao et al. analyzed 7 studies involving 781 patients who were treated with nitroprusside [12]. They assessed TFG < 3, STR, and MACEs, and their results were consistent with our findings. However, this previous meta-analysis included a retrospective study, which may have introduced bias. Our results included all RCTs to date with 4 additional summarized trials [49, 56, 57, 64]. Moreover, our meta-analysis expands previous evidence by demonstrating that the intracoronary administration of nitroprusside significantly increased LVEF after PPCI compared to standard care. We also found that nitroprusside was inferior to anisodamine for improving STR.

Adenosine is an endogenous nucleoside that modulates numerous physiological processes, such as antagonizing platelets and neutrophils, reducing calcium overload and oxygen free radicals, and inducing vasodilation [7]. Two recent meta-analyses have evaluated the role of adenosine in patients with STEMI undergoing PPCI [8, 9]. Our findings are consistent with the results of these 2 previous meta-analyses, which showed that adenosine use was associated with fewer occurrences of TFG < 3 and more occurrences of STR. However, we failed to observe the significant improvement of LVEF and benefit of the MACE endpoint for adenosine. The reason for these conflicting results may be that we included new trials [44, 49]. In the well-conducted REFLO-STEMI trial, Nazir et al. concluded that adenosine did not reduce the infarct size or NR [49]. Furthermore, they found that there were significantly worse outcomes for the adenosine group than for the control, mainly due to an excess of heart failure events. Although the findings were hypothesis generating and difficult to explain, the new data challenge the role of adenosine in PPCI and should be re-evaluated in a meta-analysis. Our updated meta-analysis showed that adenosine had no benefits in terms of LVEF and MACE endpoints, but adenosine use increased the reperfusion indices without having a harmful effect on cardiovascular outcomes other than AEs. On the basis of our results, it is still necessary to perform larger well-powered studies to definitively assess the role of adenosine in this clinical scenario.

Diltiazem and verapamil, two non-dihydropyridine calcium channel blockers, have been shown to produce endothelium-independent vasodilation and reduce calcium overload within intracellular compartments [72]. Wang et al. pooled 8 RCTs with 494 participants and found that verapamil or diltiazem was associated with a significantly improved TFG and reduced incidence of MACEs [10]. In this meta-analysis, however, verapamil and diltiazem were classified as the same group and the three included trials evaluated the effect of oral diltiazem, which might affect the results. In the current study, we separately analyzed the role of intracoronary verapamil or diltiazem. Our results based on RCTs suggest that diltiazem adjunctive therapy did not improve any outcomes studied in patients with STEMI. Although verapamil had a beneficial effect on coronary flow after PPCI, it was not associated with consistent advantages on other outcomes (STR, LVEF, and MACEs).

Nicorandil is a hybrid of nitrates and an adenosine triphosphate-sensitive potassium channel opener [73]. The mechanisms for the beneficial actions of nicorandil have been postulated, including dilation of resistance arteries, reduction of reactive oxygen species production in mitochondria, and attenuation of polymorphonuclear leukocytes activation during ischemia/reperfusion [73]. Recent meta-analysis has indicated that nicorandil was associated with improvement of coronary flow and LVEF in patients with STEMI undergoing PPCI [11]. However, the results could have been confounded by the intracoronary and intravenous administrations of nicorandil. Results of our meta-analysis showed that intracoronary nicorandil therapy leads to improvement in TFG and STR after PPCI. Unfortunately, this did not translate into significant improvement in cardiac function and clinical outcomes.

As a selective adrenoceptor blocker, urapidil may help attenuate the vasoconstrictive tendency of the coronary circulation observed after PPCI [74]. Only 2 RCTs compared urapidil with a control, and urapidil improved ventricular function in both studies. In our network meta-analysis, urapidil did not show any benefits in TFG, STR, and MACEs. For LVEF, the result of our pairwise meta-analysis showed significant improvement, whereas the result of the network meta-analysis indicated a favorable trend when intracoronary urapidil was used during PPCI. However, the relatively small sample size (49 patients) can make it difficult to interpret the data. The results for urapidil should be interpreted with caution.

It is clinically relevant to further investigate the effect of confounding factors such as age, the time to reperfusion, and duration of follow-up on the intervention effect. Ageing has been shown to be associated with reduced efficacy of cardioprotective therapies [75]. The total ischemic time was the major determinant of myocardial damage in patients with STEMI [6]. The present study evaluated MACEs at follow-up ranged from in-hospital to 12 months. Compared to studies with a longer follow-up period, those with shorter follow-up period may be inadequate for determining the differences in MACEs. Network meta-regression can be used to estimate interactions of treatment with study-level characteristics when treatment effects are heterogeneous [23]. Our meta-regression analyses suggested that the results of network meta-analysis were not confounded by age, the time to reperfusion, and duration of follow-up. However, more studies are needed to confirm these results of meta-regression analyses. Additionally, large, well-designed RCTs have shown that complete revascularization was associated with a reduction of MACEs compared with treatment of the culprit lesion only in patients with STEMI and multivessel disease [76]. This reduction was mainly driven by fewer repeat revascularizations, because all-cause mortality and non-fatal reinfarction did not differ between groups. In the present meta-analysis, the median proportion of patients with multivessel disease across 14 included studies was 52%. However, few of included studies provided data about the proportion of patients undergoing complete revascularization. Therefore, we did not perform meta-regression analysis to adjust the results of network meta-analysis for complete revascularization. We suggest that future research should investigate the impact of complete revascularization on the intervention effect. Epinephrine has beta-2 receptor agonist properties leading to the potent coronary vasodilator effect and beta-1 agonist properties mediating chronotropic and inotropic effects on the heart. Pilot studies have shown that the intracoronary administration of epinephrine reversed refractory NR in patients with STEMI [77]. We did not evaluate the safety and efficacy of epinephrine in the present study due to a lack of relevant RCTs. Additional data from RCTs are warranted to assess the effect of epinephrine on NR during PPCI.

A main strength of the present meta-analysis is that it provides the most comprehensive analysis to date of the likelihood of a range of adjunctive pharmacotherapies to prevent or reduce NR. Our results are based on mixed comparisons of multiple treatments and report treatment rankings for 7 types of intracoronary drugs. The findings from our meta-analysis can offer positive evidence regarding the use of anisodamine as an adjunct to PPCI, and pave the way for further RCTs to confirm this beneficial role of anisodamine in patients with STEMI.

Our study has several limitations. First, the present meta-analysis is based on the data of existing publications, and we could not fully assess the potential influences of comorbidities and cardiovascular medications. Second, we did not include other measures of myocardial perfusion, such as the corrected TIMI frame count, myocardial blush grade, and TIMI myocardial perfusion grade. These indices are not commonly used in clinical practice and were unavailable in most of the included studies. TFG and STR, classical indicators of reperfusion, are closely related to short-term and long-term clinical outcomes of patients with STEMI [4, 5]. Third, drug protocols varied across the eligible studies. It was difficult for us to ascertain the optimal drug protocols. Fourth, the sample size in each study was relatively small, and the CI for certain outcomes was wide because of low event rates or the absence of events. Finally, the quality of trials included in our analysis was not high.

## Conclusions

The present network meta-analysis, combining both direct and indirect evidences, showed that the intracoronary administration of anisodamine could improve myocardial reperfusion, cardiac function, and clinical outcomes in patients with STEMI undergoing PPCI. However, due to the limited quality and quantity of the included studies, the finding that anisodamine was a useful adjunct to reperfusion therapy should be viewed as hypothesis generating rather than conclusive. More rigorous RCTs are needed to verify the role of this inexpensive and well-tolerated regimen.

## Additional file

Additional file 1: Table S1. Subgroup analysis for select based on the type of control group across trials. **Figure S1.** Summary forest plot table for the outcome of major adverse cardiovascular events during short term follow-up (≤ 4.5 months). **Figure S2.** Summary forest plot table for the outcome of major adverse cardiovascular events during long-term follow-up (> 4.5 months). **Figure S3.** Summary forest plot table for the outcome of major adverse cardiovascular events defined as a composite of death, reincarnation or revascularization. (PDF 1005 kb)

## Abbreviations

AEs: Adverse events
LVEF: left ventricular ejection fraction
MACEs: Major adverse cardiovascular events
NR: No-reflow phenomenon
PPCI: Primary percutaneous coronary intervention
RCTs: Randomized controlled trials
STEMI: ST-elevation myocardial infarction
STR: ST-segment resolution
TFG: Thrombolysis in myocardial infarction flow grade
