# Supplementary material for: Effect of intracoronary agents on the no-reflow phenomenon during primary percutaneous coronary intervention in patients with ST-elevation myocardial infarction: a network meta-analysis
Source: BMC Cardiovasc Disord. 2018 Jan 10;18:3. doi: 10.1186/s12872-017-0722-z (PMC5763527; doi:10.1186/s12872-017-0722-z)
Supplement: Additional file 1: Table S1. — Subgroup analysis for select based on the type of control group across trials. Figure S1. Summary forest plot table for the outcome of major adverse cardiovascular events during short term follow-up (≤ 4.5 months). Figure S2. Summary forest plot table for the outcome of major adverse cardiovascular events during long-term follow-up (> 4.5 months). Figure S3. Summary forest plot table for the outcome of major adverse cardiovascular events defined as a composite of death, reincarnation or revascularization. (PDF 1005 kb) [file 12872_2017_722_MOESM1_ESM.pdf]

Table S1. Subgroup analysis for select outcomes based on the type of control group across trials.

Comparators	References	Outcomes (network odds ratio with 95% CI)			
		TFG < 3	STR	LVEF[#]	MACEs
Placebo					
	Anisodamine	3.66 (0.97-13.67)	0.35 (0.10-1.28)	-4.85 (-12.21 to 2.53)	4.96 (1.26-21.15)
	Nitroprusside	1.48 (0.77-3.02)	0.69 (0.36-1.19)	-5.25 (-10.89 to 0.47)	1.65 (0.92-3.05)
	Adenosine	1.70 (1.15-2.68)	0.54 (0.35-0.76)	-0.91 (-3.71 to 1.98)	1.59 (1.15-2.24)
	Diltiazem	0.86 (0.11-5.96)	1.20 (0.19-7.38)	3.09 (-7.87 to 14.26)	1.63 (0.22-11.41)
	Verapamil	6.20 (1.99-23.21)	0.69 (0.23-1.82)	-3.15 (-7.61 to 1.56)	1.31 (0.58-3.00)
	Nicorandil	-	-	-	-
	Urapidil	0.30 (0.00-20.00)	0.39 (0.07-2.06)	-3.84 (-9.06 to 1.42)	0.97 (0.02-35.26)
Conventional PPCI alone					
	Anisodamine	4.31 (1.31-13.88)	0.27 (0.08-0.99)	-6.00 (-8.48 to -3.90)	8.52 (2.38-34.98)
	Nitroprusside	2.27 (1.18-4.31)	0.82 (0.23-2.33)	0.47 (-2.93 to 2.61)	1.23 (0.37-3.56)
	Adenosine	1.92 (0.59-6.29)	0.86 (0.29-2.53)	1.50 (-1.26 to 3.67)	0.19 (0.02-1.67)
	Diltiazem	1.98 (0.66-5.75)	0.67 (0.19-2.26)	-0.22 (-3.49 to 2.96)	2.64 (0.66-11.53)
	Verapamil	3.00 (1.24 -7.17)	0.75 (0.23-2.54)	-1.25 (-3.82 to 1.10)	1.71 (0.40-7.65)
	Nicorandil	3.34 (1.50-7.96)	0.28 (0.08-0.97)	0.88 (-1.83 to 2.79)	1.50 (0.62-4.14)
	Urapidil	-	-	-	-

TFG, TIMI flow grade; STR, ST-segment resolution; LVEF, left ventricular ejection fraction; MACEs, major adverse cardiovascular events; PPCI, primary percutaneous coronary intervention. [#] Values are expressed as mean difference with 95% CI.

References			H−H Trials	Odds Ratio (95% CrI)
anisodamine (SUCRA =90%)	nicorandil	SUCRA = 65%	1	2.11 (0.53 to 8.64)<br>2.08 (0.18 to 24.51)
	diltiazem	= 62%	0	2.15 (0.30 to 13.77)<br>NA
	adenosine	= 55%	0	2.72 (0.77 to 9.66)<br>NA
	nitroprusside	= 37%	0	3.59 (0.88 to 15.55)<br>NA
	urapidil	= 37%	0	4.95 (0.08 to 214.60)<br>NA
	verapamil	= 35%	0	3.78 (0.88 to 16.09)<br>NA
	control	= 19%	2	4.66 (1.49 to 15.36)<br>4.64 (0.65 to 35.92)
nicorandil	diltiazem		0	1.01 (0.14 to 6.26)<br>NA
	adenosine		0	1.29 (0.42 to 4.06)<br>NA
	nitroprusside		0	1.71 (0.48 to 6.55)<br>NA
	urapidil		0	2.35 (0.04 to 92.89)<br>NA
	verapamil		0	1.78 (0.46 to 7.03)<br>NA
	control		4	2.21 (0.82 to 6.31)<br>2.28 (0.61 to 9.73)
diltiazem	adenosine		0	1.27 (0.27 to 7.08)<br>NA
	nitroprusside		0	1.70 (0.33 to 9.94)<br>NA
	urapidil		0	2.27 (0.03 to 114.00)<br>NA
	verapamil		1	1.76 (0.39 to 9.03)<br>1.55 (0.24 to 9.91)
	control		1	2.17 (0.51 to 11.53)<br>2.13 (0.36 to 12.51)
adenosine	nitroprusside		1	1.32 (0.58 to 3.27)<br>1.43 (0.54 to 3.78)
	urapidil		0	1.79 (0.03 to 66.75)<br>NA
	verapamil		0	1.39 (0.49 to 3.74)<br>NA
	control		6	1.71 (1.08 to 2.89)<br>1.75 (0.86 to 4.21)
nitroprusside	urapidil		0	1.36 (0.02 to 52.39)<br>NA
	verapamil		1	1.05 (0.32 to 3.18)<br>0.48 (0.04 to 5.63)
	control		1	1.30 (0.56 to 2.94)<br>1.57 (0.68 to 3.63)
urapidil	verapamil		0	0.76 (0.02 to 45.27)<br>NA
	control		1	0.96 (0.03 to 51.65)<br>1.00 (0.06 to 16.86)
verapamil	control		2	1.24 (0.51 to 3.14)<br>1.11 (0.20 to 6.75)

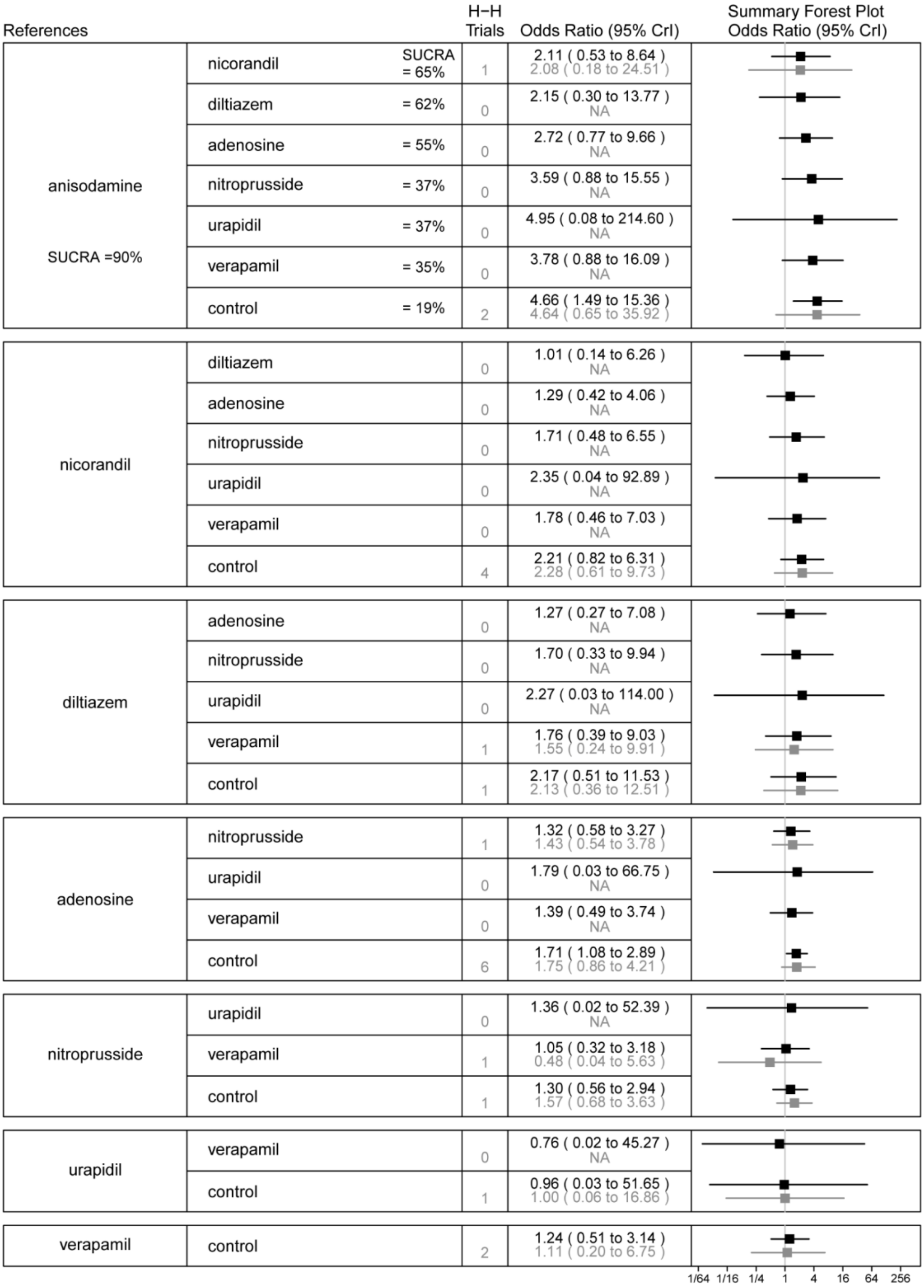

Fig. S1 Summary forest plot table for the outcome of major adverse cardiovascular events during short-term follow-up (≤4.5 months). H-H trials, head-to-head trials; black text, network meta-analysis results; grey text, pairwise meta-analysis results; CrI, credible interval; SUCRA, the surface under the cumulative ranking curve; Interventions are displayed by SUCRA percentages; Each intervention in the second column was compared with the intervention listed in the first column.

References			H−H Trials	Odds Ratio (95% CrI)
anisodamine<br>SUCRA =97%	diltiazem	SUCRA = 77%	1	3.00 (0.48 to 21.98)<br>2.84 (0.83 to 9.70)
	nitroprusside	= 64%	0	13.62 (1.00 to 510.73)<br>NA
	verapamil	= 36%	0	28.41 (1.45 to 1278.04)<br>NA
	control	= 30%	1	32.00 (2.74 to 1060.02)<br>21.08 (2.42 to 183.34)
	adenosine	= 26%	0	34.41 (2.80 to 1216.02)<br>NA
	nicorandil	= 19%	0	47.78 (2.31 to 2419.00)<br>NA
diltiazem	nitroprusside		0	4.51 (0.16 to 262.21)<br>NA
	verapamil		0	9.46 (0.25 to 665.80)<br>NA
	control		0	10.66 (0.42 to 556.92)<br>NA
	adenosine		0	11.43 (0.45 to 645.18)<br>NA
	nicorandil		0	16.06 (0.40 to 1200.02)<br>NA
nitroprusside	verapamil		0	2.09 (0.32 to 13.25)<br>NA
	control		4	2.37 (0.94 to 5.66)<br>2.19 (0.60 to 7.78)
	adenosine		1	2.51 (0.95 to 7.24)<br>2.90 (0.88 to 9.54)
	nicorandil		0	3.52 (0.48 to 23.72)<br>NA
verapamil	control		1	1.15 (0.21 to 6.00)<br>1.00 (0.17 to 5.98)
	adenosine		1	1.22 (0.24 to 6.60)<br>1.33 (0.24 to 7.28)
	nicorandil		0	1.69 (0.15 to 18.09)<br>NA
control	adenosine		7	1.06 (0.58 to 2.24)<br>1.06 (0.48 to 2.79)
	nicorandil		1	1.48 (0.26 to 8.25)<br>1.45 (0.54 to 3.89)
adenosine	nicorandil		0	1.40 (0.20 to 8.22)<br>NA

Summary Forest Plot
Odds Ratio (95% CrI)

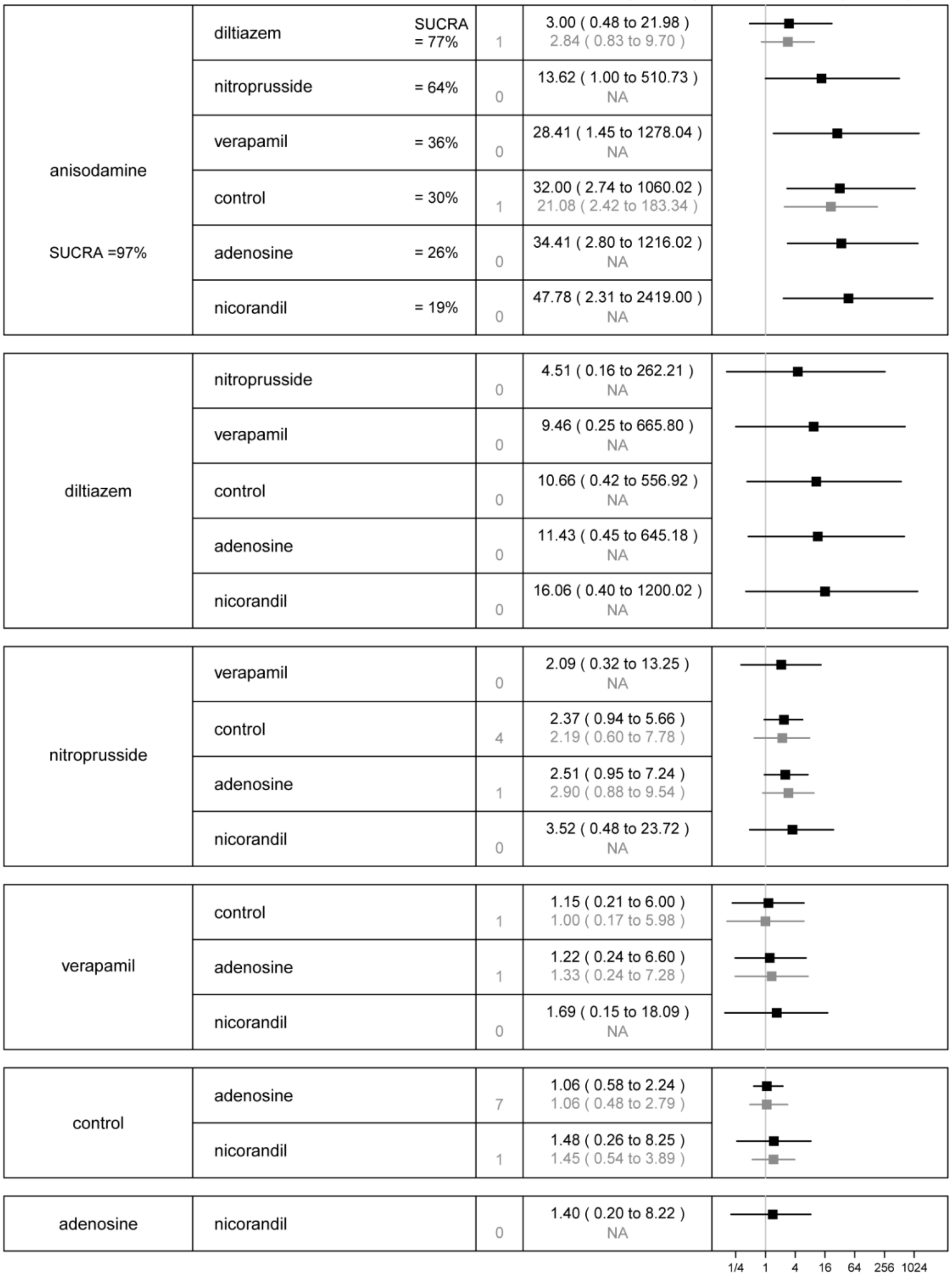

Fig. S2 Summary forest plot table for the outcome of major adverse cardiovascular events during long-term follow-up (>4.5 months). H-H trials, head-to-head trials; black text, network meta-analysis results; grey text, pairwise meta-analysis results; CrI, credible interval; SUCRA, the surface under the cumulative ranking curve; Interventions are displayed by SUCRA percentages; Each intervention in the second column was compared with the intervention listed in the first column.

References		SUCRA	H−H Trials	Odds Ratio (95% CrI)
anisodamine (SUCRA =88%)	nitroprusside	SUCRA = 80%	0	1.31 (0.19 to 9.67)<br>NA
	nicorandil	= 59%	1	2.36 (0.44 to 14.16)<br>2.08 (0.18 to 24.51)
	diltiazem	= 52%	1	2.69 (0.70 to 11.32)<br>2.84 (0.83 to 9.70)
	verapamil	= 37%	0	4.11 (0.57 to 30.21)<br>NA
	control	= 22%	1	5.44 (1.20 to 27.51)<br>3.26 (0.32 to 33.62)
	adenosine	= 13%	0	7.78 (1.01 to 65.27)<br>NA
nitroprusside	nicorandil		0	1.79 (0.34 to 9.55)<br>NA
	diltiazem		0	2.01 (0.30 to 13.52)<br>NA
	verapamil		0	3.11 (0.43 to 22.72)<br>NA
	control		2	4.10 (1.24 to 15.14)<br>4.16 (0.75 to 25.65)
	adenosine		0	5.77 (0.97 to 38.75)<br>NA
nicorandil	diltiazem		0	1.14 (0.20 to 6.29)<br>NA
	verapamil		0	1.70 (0.26 to 11.72)<br>NA
	control		3	2.29 (0.74 to 7.49)<br>2.33 (0.53 to 11.10)
	adenosine		0	3.27 (0.57 to 20.09)<br>NA
diltiazem	verapamil		1	1.52 (0.29 to 8.61)<br>1.55 (0.24 to 9.91)
	control		1	2.03 (0.50 to 8.84)<br>2.13 (0.36 to 12.51)
	adenosine		0	2.88 (0.41 to 21.40)<br>NA
verapamil	control		1	1.34 (0.28 to 6.42)<br>1.38 (0.28 to 6.68)
	adenosine		0	1.89 (0.24 to 15.06)<br>NA
control	adenosine		1	1.42 (0.36 to 5.59)<br>1.40 (0.61 to 3.22)

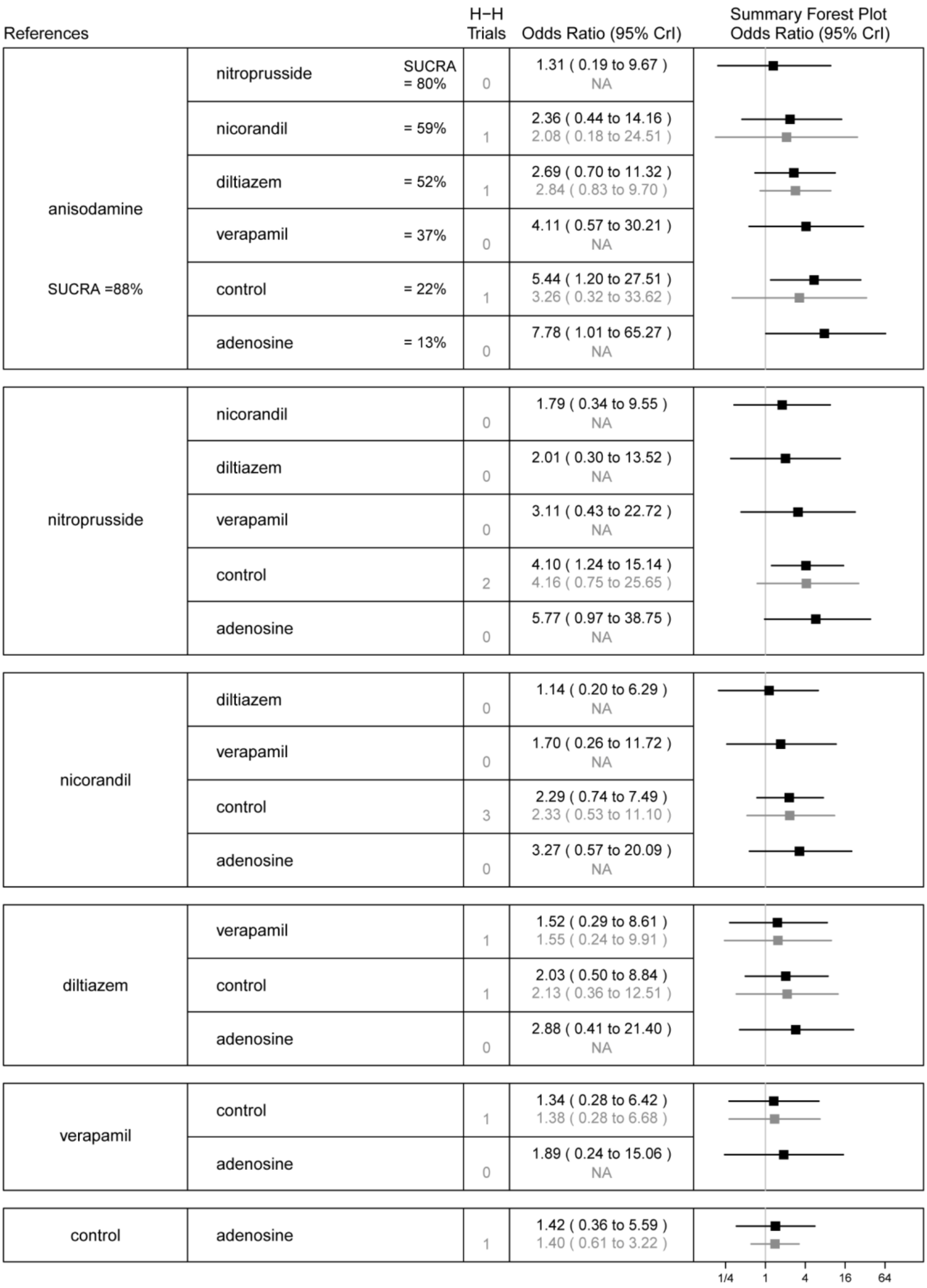

Fig. S3 Summary forest plot table for the outcome of major adverse cardiovascular events defined as a composite of death, reinfarction, or revascularization. H-H trials, head-to-head trials; black text, network meta-analysis results; grey text, pairwise meta-analysis results; CrI, credible interval; SUCRA, the surface under the cumulative ranking curve; Interventions are displayed by SUCRA percentages; Each intervention in the second column was compared with the intervention listed in the first column.
